# Extracellular volume fraction associates with long-term outcome in patients with severe symptomatic aortic stenosis: 10-year outcomes of the Regression of Myocardial Fibrosis After Aortic Valve Replacement study

**DOI:** 10.1016/j.jocmr.2026.102689

**Published:** 2026-01-15

**Authors:** Nikoo Aziminia, George D. Thornton, Jonathan Bennett, Sucharitha Chadalavada, Rebecca Kozor, Rebecca Schofield, Kush P. Patel, Iain Pierce, Peter Kellman, Rhodri Davies, Sveeta Badiani, Guy Lloyd, Mario Cortina Borja, Arantxa González, James C. Moon, Thomas A. Treibel

**Affiliations:** a Institute of Cardiovascular Science, University College London, London, United Kingdom; b Department of Cardiovascular Imaging, Barts Heart Centre, St Bartholomew’s Hospital, London, United Kingdom; c Barts Heart Centre, St Bartholomew's Hospital, Barts Health NHS Trust, London, United Kingdom; d William Harvey Research Institute, NIHR Barts Biomedical Research Centre, Queen Mary University of London, London, United Kingdom, St Bartholomew's Hospital, Barts Health NHS Trust, London, United Kingdom; e University of Sydney and Royal North Shore Hospital, Sydney, Australia; f North West Anglia NHS Foundation Trust, Peterborough, United Kingdom; g National Heart, Lung and Blood Institute, National Institutes of Health, Bethesda, Maryland, USA; h Population, Policy and Practice Research and Teaching Department, Great Ormond Street Institute of Child Health, University College London, London, United Kingdom; i Department of Cardiology and Cardiac Surgery, Clínica Universidad de Navarra and IdiSNA, Pamplona, Spain; j Program of Cardiovascular Disease, CIMA Universidad de Navarra, Clínica Universidad de Navarra and IdiSNA, Pamplona, Spain; k CIBERCV, Madrid, Spain

**Keywords:** Aortic stenosis, Left ventricular hypertrophy, Fibrosis

## Abstract

**Background:**

Diffuse fibrosis is central to the pathophysiology of aortic stenosis (AS), can be assessed using cardiovascular magnetic resonance (CMR) with extracellular volume fraction (ECV%), and is associated with mortality. The relevance of this signal to long-term prognosis remains unclear. We aim to assess predictors of long-term mortality with focus on diffuse fibrosis.

**Methods:**

Single-center prospective observational cohort study of patients with severe, symptomatic AS undergoing aortic valve replacement (AVR). Patients were assessed using echocardiography, high-sensitivity cardiac troponin T (hs-cTnT), N-terminal pro-B-type natriuretic peptide (NT-proBNP), and CMR, including T1 mapping for ECV% quantification. All-cause mortality was identified using the NHS National Spine Database. Univariable and multivariable Cox regression models were fitted to assess all-cause mortality associations.

**Result:**

One hundred and sixty-eight patients (age 72 [65–77] years, 55% [92/168] male) underwent CMR. Over a follow-up period of 9.7 (6.8–10.9) years, 76 deaths occurred. Patients who died had higher ECV% (29.9% vs 27.6%, *p* = 0.014) and greater late gadolinium enhancement (3.9% vs 2.0%, *p* = 0.013). Univariable predictors of mortality were age, atrial fibrillation (AF), left atrial area, left atrial volume, total cholesterol, triglycerides, HDL:LDL ratio, non-bicuspid aortic valve, hs-cTnT, NT-proBNP, EuroSCORE II and ECV%. On multivariable regression, age, AF and ECV% remained significant predictors of mortality, independently of sex. AIC indicated that the model with four covariates was preferable to the one also including EuroSCORE II and coronary artery disease, and this result was confirmed by a likelihood ratio test (*p*=0.387).

**Conclusions:**

In the longest follow-up cohort of T1 mapping in severe AS, we demonstrate diffuse fibrosis remains an independent predictor of long-term mortality. Integration of ECV% in baseline risk stratification should be explored further in patients with AS undergoing AVR.

## Graphical abstract

**Figure:**
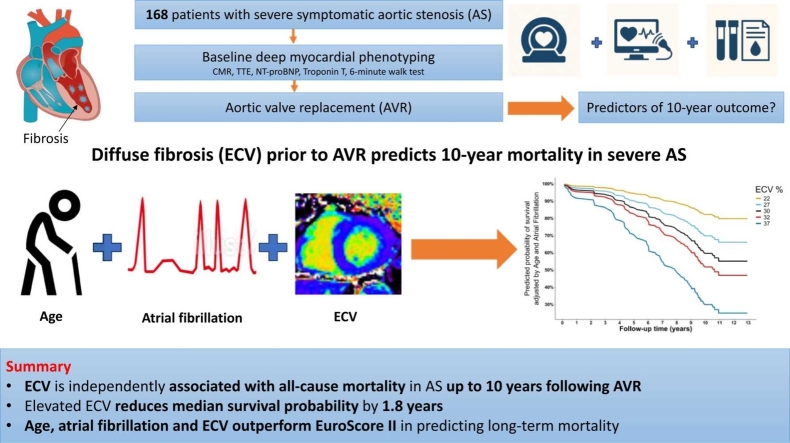

## 1 Introduction

Aortic stenosis (AS) is a disease of both valve and myocardium, characterized by progressive narrowing of the aortic valve (AV) and associated myocardial remodeling. Chronic excessive afterload stimulates this adaptive remodeling, with left ventricular hypertrophy (LVH) manifesting macroscopically, as well as myocyte and extracellular matrix remodeling microscopically. While this may initially reduce wall stress and maintain cardiac output, ultimately left ventricular decompensation, arrhythmia, heart failure (HF), and death ensue [1].

Aortic valve replacement (AVR, surgical or transcatheter) is recommended for severe AS on the basis of emergence of symptoms or left ventricular systolic impairment [2], [3]; however, this approach is increasingly recognized as requiring refinement, based on our current understanding of the natural history of AS. First, it is increasingly recognized that for some patients, this is too late [4]. Second, while severe AS patients have a high burden of myocardial fibrosis, which is associated with poor outcome [5], this is not a factor in current guidelines or clinical decision-making algorithms. Third, symptom onset is not a reliable indicator of disease severity and progression in AS [6].

Understanding the pathophysiology of AS necessitates a better understanding of the disease process within the myocardium. A key driver of decompensation and the transition from hypertrophy to HF is myocardial fibrosis. Two patterns can be appreciated: focal replacement fibrosis and diffuse interstitial fibrosis. Cardiovascular magnetic resonance (CMR) offers non-invasive characterization of both processes and correlates well with histopathological analysis [5]; focal fibrosis can be assessed using late gadolinium enhancement (LGE), whereas diffuse interstitial fibrosis can be assessed with T1 mapping, assessing the longitudinal relaxation properties of the myocardium and blood pool [7].

Over half of patients with AS undergoing AVR have focal myocardial fibrosis, which has been shown to be associated with all-cause and cardiovascular mortality up to 10 years post-AVR. But focal fibrosis is likely irreversible and therefore a less attractive target for adjuvant pharmacotherapy post-AVR compared to diffuse fibrosis, which can show some regression as early as 2 months post-AVR [8]. Extracellular volume fraction (ECV%) has been shown to associate with poor outcome in short- to medium-term follow-up [9]; however, it is unclear whether this association persists long term.

The aim of this study, therefore, was to assess the association between diffuse fibrosis and long-term mortality in severe AS, and to investigate the utility of incorporating the burden of diffuse fibrosis in the risk stratification of this patient cohort before AVR.

## 2 Methods

The Regression of Myocardial Fibrosis After Aortic Valve Replacement study was a prospective observational cohort study of patients with severe, symptomatic AS undergoing AVR at a single cardiac tertiary care referral center, University College London Hospitals NHS Foundation Trust, London, United Kingdom. As previously described [10], patients aged over 18 years with severe AS (≥2 of: AVA <1 cm^2^, mean gradient >40 mmHg, peak gradient >64 mmHg, AV velocity ratio <0.25) awaiting surgical AVR +/- coronary artery bypass grafting were prospectively recruited between January 2012 and January 2015 before pre-operative evaluation. Exclusion criteria were estimated glomerular filtration rate <30 mL/min/1.73 m^2^, CMR-incompatible devices, previous valve surgery, severe valve lesions other than AS, inability to complete protocol, and pregnancy/breastfeeding. The study was approved by the UK National Research Ethics Service (07/H0715/101), was registered with ClinicalTrials.gov (NCT02174471), and conformed with the principles of the Declaration of Helsinki, and all patients provided informed, written consent. Recruited patients underwent a comprehensive clinical assessment, including clinical history, serial blood pressure measurements, 6-minute walk test, blood sampling for N-terminal pro-B-type natriuretic peptide (NT-proBNP) and high-sensitivity cardiac troponin T (hs-cTnT), transthoracic echocardiography, electrocardiography, and CMR. Patients underwent myocardial biopsy with Congo red staining to assess for the presence of amyloidosis [11].

## 3 Cardiovascular magnetic resonance

CMR was performed at 1.5T (Magnetom Avanto, Siemens Medical Solutions, Erlangen, Germany), using a standard clinical scan protocol to assess left ventricular (LV) structure, function, and myocardial tissue characterization, as previously described [12]. The protocol included T1 mapping, LGE imaging, and ECV%.

T1 mapping (by shortened MOdified Look-Locker Inversion recovery) [13] was performed before and after gadolinium contrast (0.1 mmol/kg of Gadoterate meglumine [gadolinium-DOTA, marketed as Dotarem, Guerbet S.A., Villepinte, France]), as previously described [10]. Both pre- and post-contrast, three short-axis slices were acquired through the base, mid, and apical left ventricle. Endocardial and epicardial contours were drawn with a 10% offset to calculate the myocardial T1 pre- and post-contrast. ECV% was calculated manually using hematocrit derived from venous blood sampling on the day of scanning. American Heart Association segmentation of the slices enabled exclusion of segments with infarct-related LGE. As all patients with other cardiomyopathies and amyloidosis demonstrated on Congo red staining were excluded, non-infarct-related LGE was attributed to diffuse fibrosis in the context of AS.

ECV% was derived by manual calculation using hematocrit from venous blood sampling at the time of CMR. LGE quantification was performed using a short-axis LGE stack covering the left ventricle, using a five-standard deviation threshold for semi-automatic detection of regions of focal scar. All CMR post-processing and analysis were performed using CVI42 software version 5.1.2 (Circle Cardiovascular Imaging, Calgary, Alberta, Canada).

## 4 Echocardiography

Transthoracic echocardiography was performed using a GE Vivid E9 system (GE Healthcare, Wauwatosa, Wisconsin) with a 4-MHz transducer, to assess diastolic function and Doppler assessment of valve area and velocities, in line with the guidelines of the American Society of Echocardiography and the European Society of Echocardiography [14].

## 5 Statistical methods

The statistical methods can be found in the Supplementary Material.

## 6 Results

One hundred and sixty-eight patients with severe symptomatic AS undergoing AVR were included in the final study cohort, baseline characteristics for whom are described in Table 1. Median age was 72 years (interquartile range [IQR] 12.2 years); 92 patients (55%, 92/168) were male with a median AV area index of 0.38 cm^2^/m^2^ (0.31, 0.47) and mean gradient of 47.0 mmHg (38.9–55.6). One hundred and sixty-one patients (96%, 161/168) had ECV% data available; 167 (99%, 167/168) had LGE data available, of whom 29 (17.3%, 29/167) had infarct-pattern LGE. Twenty-two patients (13%, 22/168) had atrial fibrillation (AF) at baseline. One hundred and sixty-four (97.6%, 164/168) underwent surgical AVR and 4 (2.4%, 4/168) underwent transcatheter aortic valve implantation (TAVI). Median time from baseline CMR to AVR was 33 days (IQR 57.5). Over a median follow-up period of 9.7 years (6.8–10.9), a total of 76 (45%, 76/168) deaths occurred. Cause of death was available for 62 patients (82%, 62/168), of whom 18 (29%, 18/168) had a CV cause ascribed. Median survival was 9.7 [6.8–10.9] years.

**Table 1:** Baseline characteristics of patients categorized according to occurrence of mortality.

Characteristic	Overall (n = 168)^a^	All-cause mortality		*p-*value
		Yes (n = 76)	No (n = 92)	
Age	72 (64–77)	76 (68–80)	68 (62–75)	**<0.001**
Male sex	92 (54.8%)	44 (57.9%)	48 (52.2%)	0.458
BSA, m^2^	1.88 (1.73–2.04)	1.91 (1.72–2.04)	1.87 (1.73–2.06)	0.975
6-minute walk test distance, m	458±188	417±176	486±192	**0.044**

*Type of valve*				
Tricuspid	118 (70.2%)	65 (85.5%)	53 (57.6%)	**<0.001**
Bicuspid	49 (29.2%)	11 (14.5%)	38 (41.3%)	
Unicuspid	1 (0.6%)	0 (0.0%)	1 (1.1%)	

*Comorbidities*				
AF	22 (13.1%)	18 (23.7%)	4 (4.3%)	**<0.001**
HTN	125 (77.2%)	60 (80.0%)	65 (74.7%)	0.424
Type 2 diabetes	33 (19.6%)	17 (22.7%)	16 (18.0%)	0.508
Known CAD	50 (20.1%)	29 (38.7%)	21 (23.1%)	**0.029**
EuroSCORE II	1.49 (1.01–2.44)	1.74 (1.11–3.05)	1.36 (0.96–2.21)	**0.008**
NYHA class II–IV	138 (86.8%)	63 (86.3%)	75 (87.2%)	0.866

*Treatment*				
ACE inhibitor or ARB	71 (43.0%)	33 (44.0%)	38 (42.2%)	0.818
Aspirin	73 (44.2%)	31 (41.3%)	42 (46.7%)	0.492
Beta blocker	56 (33.9%)	26 (34.7%)	30 (33.3%)	0.857
Statin	101 (61.2%)	47 (62.7%)	54 (60.0%)	0.726

*Blood tests*				
NT-proBNP (pg/mL)	70.5 (29.0–238.3)	108.5 (44.3–313.3)	42.5 (23.8–135.5)	**<0.001**
Hs-cTnT (ng/L)	14.0 (9.0–20.0)	16.0 (13.0–25.0)	12.0 (7.0–18.0)	**<0.001**
Total cholesterol	4.7±1.2	4.4±1.0	4.9±1.3	**0.032**
Serum triglycerides	1.6±0.8	1.4±0.6	1.7±0.8	**0.014**
HDL:LDL ratio	3.4±1.2	3.0±0.9	3.6±1.3	**0.007**

*Echocardiography*				
AVA (cm^2^)	0.72 (0.55–0.90)	0.76 (0.53–0.98)	0.70 (0.57–0.89)	0.596
AVAi (cm^2^/m^2^)	0.39 (0.31–0.47)	0.40 (0.30–0.49)	0.37 (0.31–0.45)	0.268
AV peak velocity (m/s)	4.32 (4.01–4.70)	4.26 (4.00–4.66)	4.45 (4.10–4.80)	0.085
AV MG (mmHg)	47.0 (38.9–55.6)	46.1 (36.0–53.2)	48.0 (40.0–57.0)	0.101

*Cardiac magnetic resonance*				
LVEDVi (mL/m^2^)	63.1 (53.2–77.5)	63.3 (53.1–73.3)	62.8 (53.4–77.9)	0.881
LVESVi (mL/m^2^)	15.0 (10.4–25.8)	17.4 (11.9–25.6)	14.0 (9.9–26.1)	0.200
LVSVi (mL/m^2^)	44.9±10.5	44.1±9.3	45.5±11.4	0.429
LVMi (g/m^2^)	83.3 (69.2–106.0)	84.1 (69.2–107.8)	82.9 (69.4–104.7)	0.863
LVEF (%)	73.9 (63.4–80.9)	71.4 (62.3–78.9)	75.5 (63.4–81.6)	0.174
Mass volume ratio	1.34 (1.14–1.53)	1.37 (1.13–1.61)	1.32 (1.16–1.49)	0.622
GLS (%)	-19.3 (-13.6 to -29.3)	−19.2 (−12.4 to 28.4)	−19.5 (−13.7 to 29.3)	0.827
RVEDV index (mL/m^2^)	59.2 (49.3–70.0)	60.7 (50.0–71.3)	57.7 (48.9–69.1)	0.377
RVESV index (mL/m^2^)	17.5 (14.2–24.2)	18.7 (15.0–25.5)	17.2 (14.2–23.1)	0.170
RVEF (%)	68.6 (62.1–73.9)	67.8 (61.7–71.7)	69.9 (62.2–74.2)	0.167
LAAi (cm/m^2^)	13.0 (11.1–15.3)	13.3 (11.1–15.9)	12.5 (11.0–14.9)	0.179
LAVi (mL/m^2^)	56.1 (40.1–66.5)	57.6 (41.6–75.9)	48.3 (38.2–59.2)	**0.022**
ECV (%)	28.5 (26.5–30.8)	29.7 (27.3–31.1)	27.7 (26.1–30.2)	**0.014**
Cell volume (mL)	115.3 (87.0–139.2)	109.6 (90.3–135.0)	117.2 (83.3–139.2)	0.911
Matrix volume (mL)	44.5 (33.6–55.3)	43.7 (34.4–55.1)	44.7 (32.2–55.3)	0.468
LGE 5 SD (%)	2.8 (1.0–6.1)	3.9 (1.0–8.0)	2.0 (1.0–4.5)	**0.013**

*BSA* body surface area, *AF* atrial fibrillation, *HTN* hypertension, *CAD* coronary artery disease, *ACE* angiotensin converting enzyme, *ARB* angiotensin receptor blocker, *NT-proBNP* N-terminal pro-brain natriuretic peptide, *Hs-cTnT Ln* high-sensitivity troponin T (log transformed), *AV* aortic valve, *AVA* aortic valve area, *AVAi* indexed aortic valve area, *AV MG* aortic valve mean gradient, *LVEDVi* indexed left ventricular end diastolic volume, *LVESVi* indexed left ventricular end systolic volume, *LVSVi* indexed left ventricular stroke volume, *LVMi* indexed left ventricular mass, *LVEF* left ventricular ejection fraction, *GLS* global longitudinal strain, *RVEDV* right ventricular end diastolic volume, *RVESV* right ventricular end systolic volume, *RVEF* right ventricular ejection fraction, *LAAi* indexed left atrial area, *ECV%* extracellular volume fraction, *LGE* late gadolinium enhancement, *HDL* high-density lipoprotein, *LDL* low-density lipoprotein, *IQR* interquartile range, *SD* standard deviation, *LAVi* left atrial volume index, *NYHA* New York Heart Association

Bold denotes 2-sided *p* values of statistical significance (<0.05).

Data are numbers (%) of cases, means +/- standard deviaton, or medians (interquartile range).

a Median (IQR); N (%); mean ± SD

There were no significant differences in sex distribution or body surface area between patients who died and those who survived. Tricuspid AV morphology was more common among those who died than bicuspid valves (85.5% vs 57.6%; *p* < 0.001). AF and coronary artery disease were significantly more common in the mortality group (23.7% vs 4.3%, *p* < 0.001; and 38.7% vs 23.1%, *p* = 0.029, respectively). EuroSCORE II was also higher among those who died (median 1.80 vs 1.32; *p* = 0.008). Patients who died achieved shorter 6-minute walk test distances than those who survived (417 ± 176 m vs 486 ± 192 m, *p* = 0.044). There were no significant differences in the prevalence of hypertension, diabetes, or New York Heart Association class II–IV symptoms between groups. Use of angiotensin converting enzyme (ACE) inhibitors/angiotensin receptor blockers and statins was similar between groups; however, beta blocker use was marginally lower in those who died (*p* = 0.032). In terms of cardiac biomarkers, NT-proBNP and hs-cTnT were significantly elevated in the mortality group (*p* < 0.001 for both). Echocardiographic parameters of AS severity did not significantly differ between groups. On CMR, patients who died had higher ECV% (29.9% vs 27.6%, *p* = 0.014) and greater LGE burden (3.9% vs 2.0%, *p* = 0.013). Left ventricular volumes, function, mass index, and strain parameters did not differ significantly by outcome (Table 1).

55% (92/168) of patients in this cohort were male. Differences in patterns of cardiac remodeling according to sex were observed as previously reported from this dataset [15]. Among the patients who died, there was no significant difference in ECV% between male and female patients (30.1 [27.0, 31.8] vs 29.7 [27.4, 31.1], *p* = 0.605)*.*

### 6.1 Predictors of mortality

Upon univariable analysis (Supplementary Table 1), significant predictors of mortality were age, AF, left atrial area, left atrial volume, hs-cTnT, NT-proBNP, total cholesterol, triglycerides, HDL:LDL ratio, EuroSCORE II, non-bicuspid AV, and ECV%. Patient’s sex, body surface area, 6-minute walk test distance, hypertension, coronary artery disease, concomitant coronary artery bypass grafting, AS severity by echocardiography, LV mass, matrix volume, cell volume, global longitudinal strain, and LGE were not.

Multivariable analysis carried out following imputation, model selection, and biplot to find clusters of correlated covariances, yielded age, AF, and ECV% as predictors of long-term all-cause mortality (Table 2). There were no significant interaction terms between age and other covariates.

**Table 2:** Multivariable Cox regression models for the full dataset with post-imputation estimates for predictors of mortality.

	HR	Lower	Upper	*p-*value
*A*				
Age	1.076	1.043	1.111	<0.001
Sex	1.392	0.877	2.211	0.160
AF	2.165	1.255	3.735	0.006
ECV%	1.127	1.041	1.219	0.003
				
*B*				
Age	1.075	1.041	1.110	<0.001
Sex	1.418	0.893	2.251	0.138
AF	2.009	1.152	3.505	0.014
ECV%	1.124	1.038	1.217	0.004
EuroScore II	1.081	0.954	1.225	0.225
				
*C*				
Age	1.074	1.041	1.109	<0.001
Sex	1.301	0.808	2.093	0.279
AF	2.165	1.257	3.729	0.005
ECV%	1.137	1.048	1.233	0.002
CAD	1.343	0.822	2.196	0.239
				
*D*				
Age	1.074	1.040	1.108	<0.001
Sex	1.345	0.830	2.181	0.229
AF	2.053	1.173	3.594	0.012
ECV%	1.132	1.043	1.228	0.003
EuroScore II	1.056	0.917	1.215	0.451
CAD	1.231	0.714	2.123	0.454

*AF* atrial fibrillation, *CAD* coronary artery disease, *ECV%* extracellular volume fraction, *HR* hazard ratio

A: final model; B: model including EuroScore II, C: model including CAD, and D: model including EuroScore II and CAD

Data are numbers (%) of cases, means +/- standard deviaton, or medians (interquartile range).

**Fig. 1:**
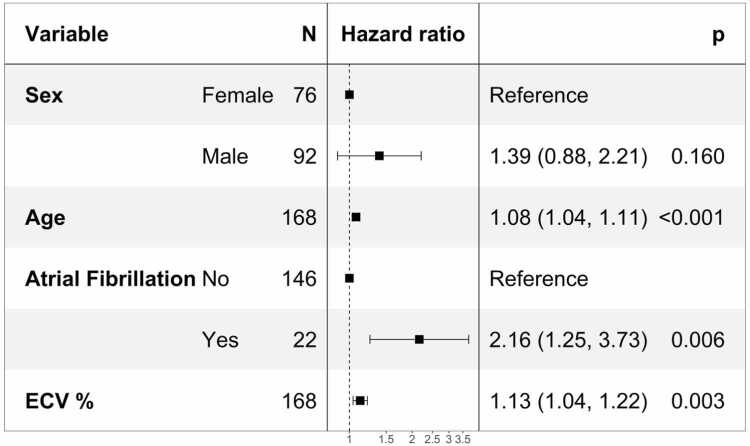
Forest plot of hazard ratios and 95% confidence intervals for all-cause mortality. *ECV%* extracellular volume fraction

Despite observing no differences in mortality by sex, we included it as a covariate in the fitted final model to estimate sex-adjusted hazard ratios which allows comparisons of our results with other analyses (Fig. 1) [16]. Each additional year of age increased mortality risk by 7.6%, AF more than doubled the risk, and each unit increase in ECV% was associated with a 12.7% higher mortality risk.

We assessed the predictive characteristics of the final model against those for a univariable model with EuroSCORE II only, and a multivariable model adding this covariate to those included in the model shown in Table 2. We observed a lower imputed-averaged Akaike’s information criterion (AIC) value for the final multivariable model (AIC = 694.3) compared to the univariable model including only EuroSCORE II (AIC = 725.5), indicating better overall model fit and parsimony and therefore demonstrating the simplest model captures the essential patterns in the data. This suggests the multivariable model using age, sex, AF, and ECV% improves accuracy of mortality risk prediction beyond EuroSCORE II alone.

We also formally tested whether the inclusion of EuroSCORE II and coronary artery disease in the model would enhance mortality prediction, using a likelihood ratio test adjusted for multiple imputation to compare the nested models with age, sex, AF, and ECV% against the model adding EuroSCORE II, coronary artery disease, and both EuroSCORE II and coronary artery disease (Table 2A). This yielded *p* = 0.227, 0.239, and 0.457, respectively, thus not rejecting the null hypothesis of equal goodness-of-fit of all four models. We observed the lowest imputed-averaged AIC value for a final multivariable model consisting of age, sex, AF, and ECV% (AIC = 694.2) compared with nested models, including EuroSCORE II (AIC = 694.9), coronary artery disease (AIC = 694.9), and both EuroSCORE II and coronary artery disease (AIC = 696.3). Based on the AIC values, the model consisting of these with sex alone demonstrated the best overall model fit and parsimony. In all four models, age, AF, and ECV% emerged as robust multivariable predictors of all-cause mortality independently of sex, EuroSCORE II, and the presence of coronary artery disease.

Finally, we performed bootstrap-based inference on 1000 samples for the three metrics (integrated discrimination improvement [IDI], net reclassification index [NRI], and *C*-statistic) defined by Uno et al. [17] and implemented in the R library survIDINRI, to assess the effects of adding EuroSCORE II and CAD to the final model with four covariates [18]. There was no evidence from the three metrics (*p* = 0.198, 0.464, 0.510) that the addition of EuroSCORE II to the final model improved mortality prediction, and the same was observed for adding CAD to the final model (*p* = 0.312, 0.555, 0.953). We also assessed the effects of adding ECV% to the model, including age, sex, and AF using the NRI, IDI, and C-statistic; this indicated a significant improvement in prediction attributable to ECV% (*p* = 0.014, 0.032, 0.020, respectively).

A sensitivity analysis was conducted by fitting the multivariable Cox proportional hazards models to the six covariates using the cohort comprising 147 patients (87.5%), who had no missing data in these covariates (Supplementary Table 2). Further sensitivity analysis was conducted using the cohort comprising 146 patients (86.9%) with no AF, by fitting multivariable Cox proportional hazards models to the same covariates with the exception of AF (Supplementary Table 3). Both analyses yielded the same conclusions as those from the models fitted using the imputed dataset (Table 2).

### 6.2 Stratification of survival according to age, AF status, and ECV%

**Fig. 2:**
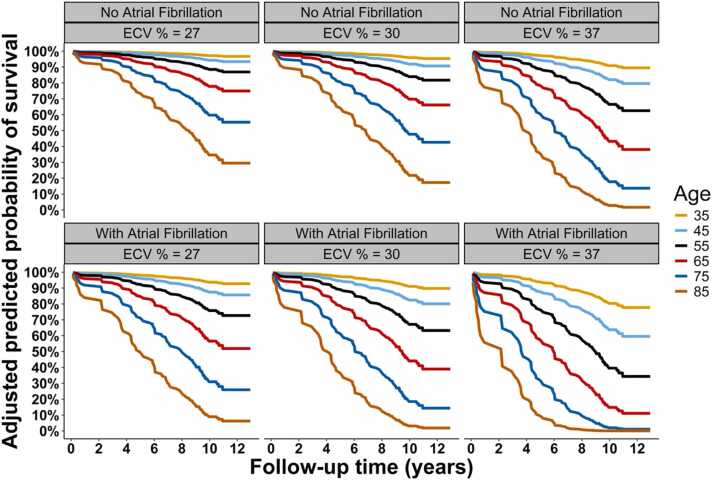
Adjusted predicted probability of survival by age, atrial fibrillation, and ECV. *ECV%* extracellular volume fraction

Increasing level of ECV% was associated with worsening survival, following adjustment for age, sex, and presence of AF (Supplementary Fig. 1). The adjusted predicted probability of survival declined progressively with increasing age irrespective of AF status and across all levels of ECV% (Supplementary Fig. 2), and also when stratified into tertiles (27%, 30%, 37%). Within each ECV% stratum, older age was associated with markedly reduced survival (Fig. 2). Additionally, the presence of AF further accentuated the risk, resulting in consistently lower survival probabilities compared to those without AF at equivalent ECV% and age levels (Fig. 2, Supplementary Fig. 3).

## 7 Discussion

Our study represents the longest follow-up of patients with severe AS undergoing AVR according to baseline in vivo assessment of myocardial structure, function, and tissue characterization. Our key findings are (1) in patients with severe AS, baseline ECV% is independently associated with all-cause mortality up to 10 years following AVR; (2) patients with elevated baseline ECV% have a 1.8-year reduction in mean survival; (3) when stratifying patients according to age group, older patients (age>75 years) in the high ECV% group experienced a steeper decline in survival probability compared with younger patients, demonstrating a compounded adverse impact of elevated ECV%, advanced age and AF on long-term survival; (4) age, AF, and baseline ECV% are independent predictors of mortality, independently of sex and the presence of coronary artery disease, with a 1% rise in ECV% resulting in a 13% increase in long-term mortality hazard; and (5) risk stratification of patients using these three parameters outperforms EuroSCORE II in assessing long-term all-cause mortality.

Current guidelines recommend AVR in the context of severe AS with evidence of LV decompensation, heralded by either onset of symptoms or impairment in LVEF. In our study, we have demonstrated an under-recognized component; diffuse scar present before intervention is associated with increased mortality hazard, which persists up to 10 years post-AVR. While EuroSCORE II is used to assess in-hospital mortality hazard following cardiac surgery and not specifically for AS, it has previously demonstrated utility in predicting mortality following TAVI [19]. Our study highlights that ECV% derived from CMR, along with clinical parameters easily assessed before intervention, provides added value in risk stratification of patients with severe AS undergoing AVR. Current guidelines do not provide guidance on pharmacotherapy in AS to target residual hypertrophy, fibrosis, or functional impairment; however, the burden of HF following AVR is increasingly recognized, contributing to early readmission and higher mortality [20], [21]. AS-specific risk stratification tools incorporating CMR-derived parameters of myocardial health are therefore needed to establish the risk of long-term residual adverse risk and to guide adjuvant medical therapy post-AVR using medications with prognostic value demonstrated in HF, such as ACE inhibitors, sodium glucose co-transporter 2 inhibitors, and MRAs.

The utility of ECV% in the prognostication of severe AS at long-term follow-up is increasingly appreciated. CT- and CMR-derived assessments of ECV% demonstrate good correlation in patients with AS and cardiac amyloidosis, both of which are increasingly utilized as tools for prognostication in AS [22]. Our previous data demonstrated that ECV% assessed using CMR is associated with myocardial health and mortality at up to 3.8 years’ follow-up, demonstrating an association with worsening left and right ventricular remodeling function, and burden of LGE according to ECV% tertiles [23]. High baseline CMR-derived ECV% was associated with greater all-cause and cardiovascular mortality, with no significant interaction by sex. In women, ECV% at baseline remained a strong predictor of all-cause mortality after multivariable adjustment [24]. More studies are required to assess the prognostic significance of ECV% derived from these techniques over long-term follow-up. The present study supports this finding and demonstrates the persistence of this effect beyond 9 years following AVR, with worsening survival according to ECV% category at baseline. Although ECV% was a strong predictor of long-term mortality, matrix volume and cell volume were not; this is likely attributable to the interaction with LV mass, which was also non-significant on univariable Cox regression and was used to calculate both variables.

Aging plays a key role in cardiac remodeling in normal loading conditions as well as in AS, influencing remodeling of the valve, aortic root, and myocardium [25]. Age-associated changes take place within the heart at the cellular and interstitial levels. Myocyte death may lead to hypertrophy of remaining myocytes, and/or development of replacement fibrosis, while activation of cardiac fibroblasts can also lead to interstitial fibrosis, irrespective of the structure and function of the valves [25]. A longitudinal study of patients aged 54–94 years with no clinical cardiovascular disease demonstrated an age-related increase in concentric LV remodeling, with a longitudinal increase in LV mass and a significant longitudinal decrease in LV volume [26]. Therefore, adverse myocardial remodeling and fibrosis may take place as patients age, which may be further accentuated in the context of AS. In the present study, we show that the elevated risk of mortality exerted by high ECV% is not uniform across all ages, with the highest mortality in the oldest patients of our cohort, a trend which has not been previously demonstrated. While AS is largely a disease of older age in the Western world, this highlights the importance of interpreting ECV% in the context of patients’ age during baseline assessment.

We also show that AF is independently predictive of all-cause mortality, doubling the risk of death. These findings support previous findings in patients with severe AS undergoing AVR, irrespective of symptom status [27]. The relationship between AF and AS may be multifactorial. AF may be associated with mitral valve and left atrial remodeling secondary to the retrograde pressure loading with AV stenosis, as captured in the echocardiography-based classification of extra-valvular damage in AS [28]. Atrial fibrosis may also play a role; a study of histological and molecular markers of fibrosis in patients with AF and sinus rhythm undergoing AVR with the same degree of AS severity showed increased fibrosis volume, collagen III gene expression, and decreased proteolytic enzyme expression, demonstrating abnormalities in collagen metabolism and thus accumulation seen histologically [29]. Whether patients with AS are more susceptible to atrial fibrosis, leading to AF, or whether the combination of AS and AF contributes to atrial fibrosis is unclear, as is the prognostic significance of atrial fibrosis. The mortality risk association of AF may be associated with concomitant cardiac abnormalities. One study of patients with moderate and severe AS demonstrated higher 10-year mortality in patients with AF irrespective of AS severity and LVEF; however, this association was lost following correction for echocardiographic markers of diastolic dysfunction [30]. Our study did not assess markers of diastolic function; however, left atrial area was a univariable predictor of mortality in AS.

Sex-specific pathophysiology of AS is increasingly studied, including both the burden and prognostic significance of myocardial fibrosis. Previous work by this group has demonstrated greater matrix volume and cell volume in men compared with women with severe AS, however no difference in ECV% [15]. The burden of ECV% by sex may differ according to AS severity; a previous study of patients with all degrees of AS severity demonstrated a higher ECV% in women than in men [31]. The prognostic significance of baseline diffuse fibrosis has been demonstrated in both sexes; baseline ECV% is associated with higher all-cause and cardiovascular mortality in severe AS at medium-term follow-up, with no interaction with sex [24]. Our findings support the notion that ECV% before AVR holds prognostic significance in both men and women, in line with existing data. There was no difference observed between the sexes, neither according to baseline ECV% nor mortality associated with high and low ECV% groups. ECV% may therefore be valuable in assessing mortality hazard in both sexes before AVR, using the same cut-off for baseline risk stratification.

Focal fibrosis measured as LGE has been shown to be a marker of poor prognosis in previous studies [32], and demonstrated the long-term prognostic significance of focal fibrosis as assessed by LGE in AS, by the presence and irrespective of pattern of fibrosis [33]. Another study reported that in patients with high-gradient, normal LVEF severe AS, this association was lost in multivariable Cox regression analysis, including ECV%, demonstrating the stronger predictive value of ECV% in detecting long-term adverse outcome [34]. LGE quantification is more subjective and error-prone than T1 mapping. These factors may explain why it did not reach significance in our study; nevertheless, LGE was associated with histological fibrosis, NT-proBNP, and hs-cTnT [5].

## 8 Limitations

Our study has a number of strengths and limitations. A key strength is the inclusion of a wide array of patients with a guideline-based indication for AVR, representing 50% of all referrals, with the inclusion of patients with hypertension, diabetes, and coronary artery disease; the study population is therefore highly representative of that encountered by the AV multidisciplinary team, supporting the translation of our findings into clinical practice. Another strength is the exclusion of patients with amyloidosis by endomyocardial biopsy; the presence of cardiac amyloidosis has been previously demonstrated in this cohort as the strongest univariable predictor of mortality [11]. Therefore, exclusion of these patients allows better appreciation of other potential predictors of mortality based on pre-AVR CMR. Limitations include the potential for selection bias; all patients had severe AS and had already been deemed appropriate candidates for predominantly surgical AVR; we therefore cannot comment on the burden of baseline fibrosis in those deemed unfit for surgical AVR based on existing surgical risk scores. Finally, although our study contributes to our understanding of the impact of baseline diffuse fibrosis on all-cause mortality, it was not sufficiently powered to comment on the association with cardiovascular mortality.

## 9 Conclusions

Diffuse fibrosis in severe AS measured as ECV% is independently associated with a greater risk of mortality at long-term follow-up of up to 10 years. High baseline ECV%, age, and AF are associated with a reduction in survival by 1.8 years and outperform EuroSCORE II in mortality prediction. This underscores the importance of incorporating CMR-derived ECV% into baseline risk stratification of patients with AS.

## Funding

T.A.T. is supported by an intermediate research fellowship from the 10.13039/501100000274British Heart Foundation (FS/19/35/34374). J.C.M. and T.A.T. are directly and indirectly supported by the University College London Hospitals NIHR Biomedical Research Centre and Biomedical Research Unit at Barts Hospital, respectively. A.G. is supported by the 10.13039/501100004587Instituto de Salud Carlos III (PI24/00844 co-financed with ERDF funds), the 10.13039/501100011033Agencia Estatal de Investigación and Horizon Europe ERA4HEALTH CARDINNOV program (PCI2024-153490), and 10.13039/100008666Fundació la Marató de TV3 (202321-31). M.C.B. was supported by the 10.13039/501100000272National Institute for Health Research (NIHR) Great Ormond Street Hospital Biomedical Research Centre.

## Author contributions

**Nikoo Aziminia:** Writing – review & editing, Writing – original draft, Visualization, Validation, Software, Project administration, Methodology, Formal analysis, Data curation. **George D. Thornton:** Writing – review & editing. **Jonathan Bennett:** Writing – review & editing. **Sucharitha Chadalavada:** Writing – review & editing, Methodology, Investigation, Data curation. **Rebecca Kozor:** Writing – review & editing, Methodology, Investigation, Data curation. **Rebecca Schofield:** Writing – review & editing, Methodology, Investigation, Data curation. **Kush P Patel:** Writing – review & editing, Software, Investigation. **Iain Pierce:** Writing – review & editing, Software, Resources. **Peter Kellman:** Writing – review & editing, Software, Resources. **Rhodri Davies:** Writing – review & editing, Methodology, Investigation, Data curation. **Sveeta Badiani:** Writing – review & editing, Methodology, Investigation, Data curation. **Guy Lloyd:** Writing – review & editing, Resources. **Mario Cortina Borja:** Writing – review & editing, Visualization, Validation, Supervision, Resources, Methodology, Formal analysis. **Arantxa González:** Writing – review & editing, Data curation. **James C. Moon:** Writing – review & editing, Supervision, Resources, Methodology, Funding acquisition, Conceptualization. **Thomas A. Treibel:** Writing – review & editing, Supervision, Software, Resources, Project administration, Methodology, Investigation, Funding acquisition, Formal analysis, Data curation, Conceptualization.

## Declaration of competing interests

The authors declare that they have no known competing financial interests or personal relationships that could have appeared to influence the work reported in this paper.

## Data Availability

Data used in the preparation of this manuscript can be made available upon reasonable request to the senior author.
