# Supplementary material for: Extracellular volume fraction associates with long-term outcome in patients with severe symptomatic aortic stenosis: 10-year outcomes of the Regression of Myocardial Fibrosis After Aortic Valve Replacement study
Source: J Cardiovasc Magn Reson. 2026 Jan 15;28(1):102689. doi: 10.1016/j.jocmr.2026.102689 (PMC13187588; doi:10.1016/j.jocmr.2026.102689)

# SUPPLEMENTARY MATERIAL

**Extracellular volume fraction associates with long-term outcome in patients with severe symptomatic aortic stenosis:**

**10-year outcomes of the RELIEF-AS Study**

***Statistical analysis***

All statistical analyses were performed using SPSS software version 22 (IBM, Armonk, New York), and R version 4.4.2 (R Foundation for Statistical Computing, Vienna, Austria). Normality was assessed using the Shapiro-Wilk test. Continuous variables were summarised with median and interquartile range as most variables were non-normally distributed; categorical variables were summarised as counts and percentages. Baseline characteristics of participants were compared between groups using χ^2^ tests for categorical variables and Wilcoxon-Mann-Whitney *U*-tests for continuous variables. Log transformation was applied to normalise the distributions of NTproBNP and hsTnT. Correlations were estimated with Spearman’s correlation coefficient. The index date was given as the date of baseline CMR. Univariable associations of all-cause were established via Kaplan-Meier survival curves. Cox proportional hazards (PH) models were fitted for all-cause mortality. In order to minimise multicollinearity, associations between covariates were assessed using a biplot from a principal component analysis. Missing values were dealt with multivariate imputation by chained equations with 30 imputations performed over all covariates and included the time to death as a predictor.[18] We fitted logistic regression models with missingness as response to assess the plausibility of the missing-at-random assumption. Model selection was based on a stepwise procedure based on averaging Akaike’s Information Criterion (AIC) over the 30 imputed datasets [19], and the final model’s estimates and their standard errors were obtained using Rubin’s rule to pool results from the imputed datasets.[20] For nested models we tested goodness-of-fit using likelihood ratio tests adjusted for multiple imputation.[21] The linearity of the relationship between mortality and covariates was assessed via likelihood ratio tests comparing a natural cubic spline function with 2 degrees of freedom, and interactions with age an all covariates in the model were tested using likelihood ratio tests. We evaluated the added value of a covariate by looking at three metrics defined over the distributions of risk scores arising from two Cox PH models: the original one, and the augmented with one extra covariate. These metrics are the integrated discrimination improvement index (IDI) which is based on area-under-the-curve statistic for the predictive survival distributions; the net classification improvement index (NRI) which measures the vertical distance between these distributions at a specified time-to-event value; and the difference of predicted medians (DPM) which measures the distance between times required by each model at the median number of events. These metrics have been previously characterised [22]; for all three, large values indicate significant improvement in the predictive power of the augmented model relative to the original one. Inference on these metrics was performed via 1000 bootstrap samples after adjusting for censoring by the inverse probability censoring weight using the survIDINRI R library.[23] A 2-sided *p*-value < 0.05 was considered statistically significant.

**Supplementary Table 1:** Univariable Cox proportional hazards regression models for of predictors of all-cause mortality fitted to complete-case data.

| **Covariate** | **HR** | **95% CI** | **χ^2^** | ***p*-value** |
| --- | --- | --- | --- | --- |
| Age | 1.075 | 1.043, 1.108 | 21.911 | **<0.001** |
| Sex | 1.304 | 0.826, 2.058 | 1.305 | 0.255 |
| BSA | 0.867 | 0.307, 2.453 | 0.072 | 0.788 |
| 6-minute walk test | 0.999 | 0.997-1.000 | 3.499 | 0.062 |
| AF | 3.098 | 1.818, 5.279 | 19.191 | **<0.001** |
| HTN | 1.216 | 0.690, 2.141 | 0.459 | 0.499 |
| CAD | 1.578 | 0.991, 2.512 | 3.753 | 0.055 |
| Concomitant CABG | 1.188 | 0.715, 1.972 | 0.443 | 0.506 |
| EuroSCORE II | 1.175 | 1.058, 1.304 | 9.327 | **0.002** |
| HsTnT (Ln) | 1.608 | 1.137, 2.274 | 7.262 | **0.007** |
| NTproBNP (Ln) | 1.411 | 1.170, 1.701 | 13.332 | **<0.001** |
| Total cholesterol | 0.745 | 0.574, 0.967 | 4.874 | **0.027** |
| Triglycerides | 0.619 | 0.407, 0.940 | 5.069 | **0.025** |
| HDL:LDL ratio | 0.696 | 0.526, 0.920 | 6.501 | **0.011** |
| AVA | 1.400 | 0.620, 3.160 | 0.655 | 0.418 |
| AV MG | 0.987 | 0.970, 1.004 | 2.321 | 0.127 |
| LAA | 1.036 | 1.002, 1.071 | 4.326 | **0.038** |
| LAV | 1.010 | 1.005, 1.016 | 12.920 | **<0.001** |
| LVEF | 0.993 | 0.979, 1.007 | 0.965 | 0.326 |
| BAV | 0.359 | 0.189, 0.681 | 10.730 | **0.002** |
| LVEDV | 1.000 | 0.995, 1.005 | 0.004 | 0.950 |
| LVESV | 1.002 | 0.996, 1.007 | 0.421 | 0.517 |
| LVM | 1.000 | 0.996, 1.004 | 0.013 | 0.911 |
| LGE 5SD | 1.037 | 0.998, 1.077 | 3.519 | 0.062 |
| ECV% | 1.113 | 1.032, 1.201 | 7.683 | **0.006** |
| Cell volume | 0.999 | 0.992, 1.005 | 0.192 | 0.661 |
| Matrix volume | 1.003 | 0.991, 1.016 | 0.285 | 0.593 |
| GLS | 1.000 | 0.945, 1.057 | 0.000 | 0.988 |

BSA = body surface area; AF = atrial fibrillation; HTN = hypertension; CAD = coronary artery disease; HsTnT (Ln) = high-sensitivity troponin T (log transformed); NTproBNP (Ln)= N-terminal pro brain natriuretic peptide (log transformed); AVA = aortic valve area; AV MG = aortic valve mean gradient; LAA = left atrial area; LVEF = left ventricular ejection fraction; BAV = bicuspid aortic valve; LVEDV = left ventricular end diastolic volume; LVESV = left ventricular end systolic volume; LVM = left ventricular mass; LGE = late gadolinium enhancement; ECV% = extracellular volume fraction; GLS = global longitudinal strain.

**Supplementary Table 2:** Sensitivity analysis in patients with complete dataset without imputation.

| **A** | **HR** | **Lower** | **Upper** | ***p-*value** |
| --- | --- | --- | --- | --- |
| Age | 1.082 | 1.046 | 1.119 | <0.001 |
| Sex | 1.265 | 0.775 | 2.067 | 0.348 |
| AF | 1.892 | 1.042 | 3.434 | 0.036 |
| ECV | 1.124 | 1.036 | 1.220 | 0.005 |
| **B** | **HR** | **Lower** | **Upper** | ***p-*value** |
| Age | 1.081 | 1.045 | 1.118 | <0.001 |
| Sex | 1.323 | 0.807 | 2.168 | 0.267 |
| AF | 1.692 | 0.915 | 3.130 | 0.094 |
| ECV | 1.123 | 1.035 | 1.219 | 0.006 |
| EuroScore II | 1.096 | 0.960 | 1.252 | 0.174 |
| **C** | **HR** | **Lower** | **Upper** | ***p-*value** |
| Age | 1.080 | 1.044 | 1.117 | <0.001 |
| Sex | 1.188 | 0.719 | 1.963 | 0.501 |
| AF | 1.893 | 1.045 | 3.428 | 0.035 |
| ECV | 1.136 | 1.044 | 1.236 | 0.003 |
| CAD | 1.340 | 0.803 | 2.238 | 0.262 |
| **D** | **HR** | **Lower** | **Upper** | ***p-*value** |
| Age | 1.080 | 1.044 | 1.117 | <0.001 |
| Sex | 1.260 | 0.752 | 2.113 | 0.380 |
| AF | 1.735 | 0.933 | 3.225 | 0.082 |
| ECV | 1.130 | 1.038 | 1.230 | 0.005 |
| EuroScore II | 1.074 | 0.925 | 1.246 | 0.351 |
| CAD | 1.201 | 0.682 | 2.116 | 0.526 |

**Supplementary Table 3:** Sensitivity analysis in patients without atrial fibrillation.

| **A** | **HR** | **Lower** | **Upper** | ***p-*value** |
| --- | --- | --- | --- | --- |
| Age | 1.087 | 1.050 | 1.126 | <0.001 |
| Sex | 1.548 | 0.908 | 2.637 | 0.108 |
| ECV | 1.116 | 1.027 | 1.214 | 0.010 |
| **B** | **HR** | **Lower** | **Upper** | ***p-*value** |
| Age | 1.085 | 1.046 | 1.126 | <0.001 |
| Sex | 1.390 | 0.809 | 2.387 | 0.233 |
| ECV | 1.108 | 1.017 | 1.206 | 0.019 |
| EuroScore II | 1.100 | 0.946 | 1.280 | 0.215 |
| **C** | **HR** | **Lower** | **Upper** | ***p-*value** |
| Age | 1.082 | 1.044 | 1.121 | <0.001 |
| Sex | 1.355 | 0.768 | 2.390 | 0.294 |
| ECV | 1.126 | 1.033 | 1.228 | 0.007 |
| CAD | 1.411 | 0.790 | 2.520 | 0.245 |
| **D** | **HR** | **Lower** | **Upper** | ***p-*value** |
| Age | 1.083 | 1.043 | 1.124 | <0.001 |
| Sex | 1.299 | 0.732 | 2.305 | 0.371 |
| ECV | 1.113 | 1.019 | 1.215 | 0.017 |
| EuroScore II | 1.096 | 0.929 | 1.294 | 0.275 |
| CAD | 1.138 | 0.601 | 2.151 | 0.692 |

***Supplementary Figure 1:*** *Adjusted predicted probabilities by ECV%, adjusted by atrial fibrillation, sex, and age.*


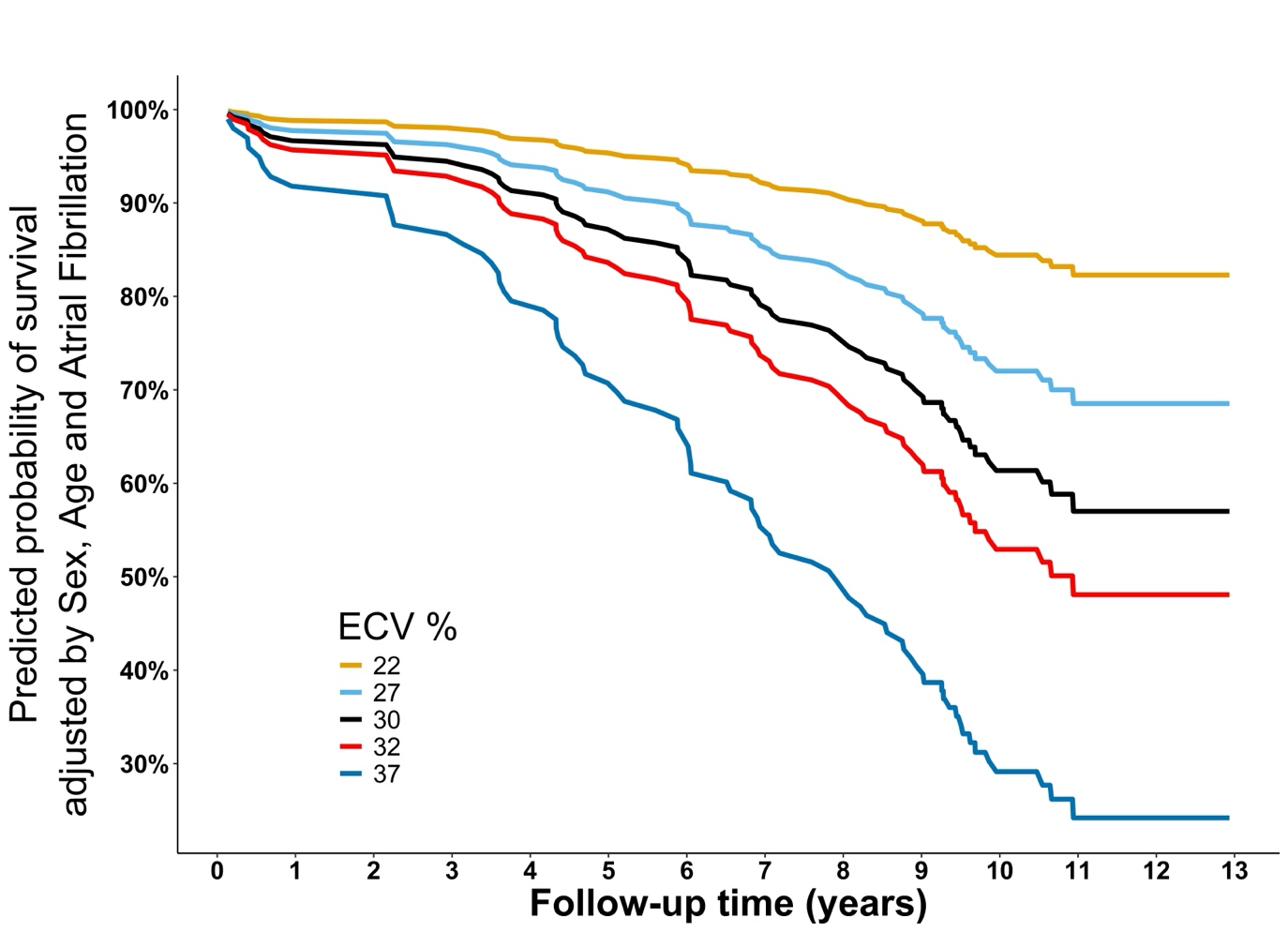


***Supplementary Figure 2:*** *Adjusted predicted probabilities by age, adjusted by atrial fibrillation, sex, and ECV%.*


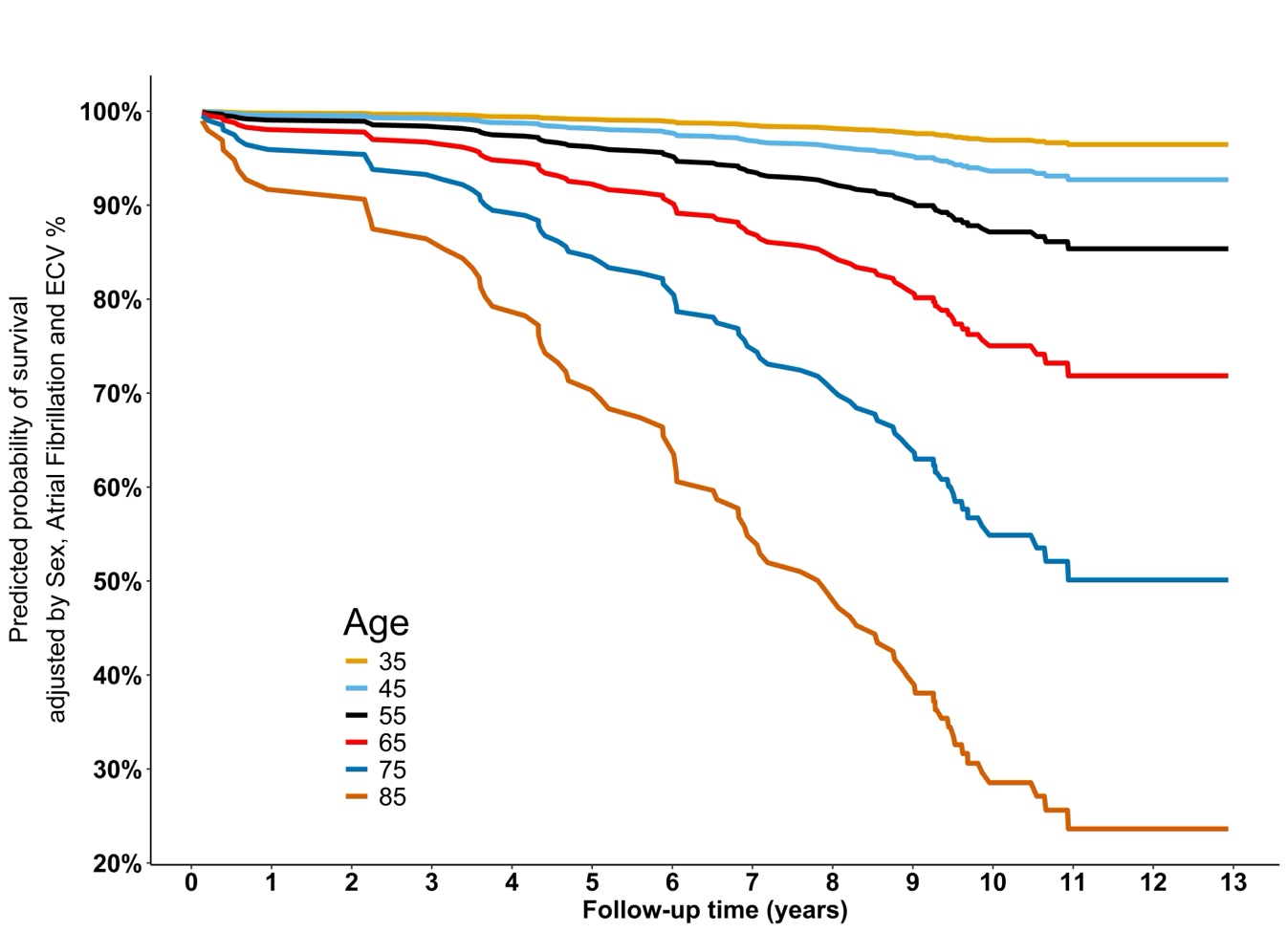


***Supplementary Figure 3:*** *Adjusted predicted probabilities by atrial fibrillation, adjusted by sex, age and ECV%.*


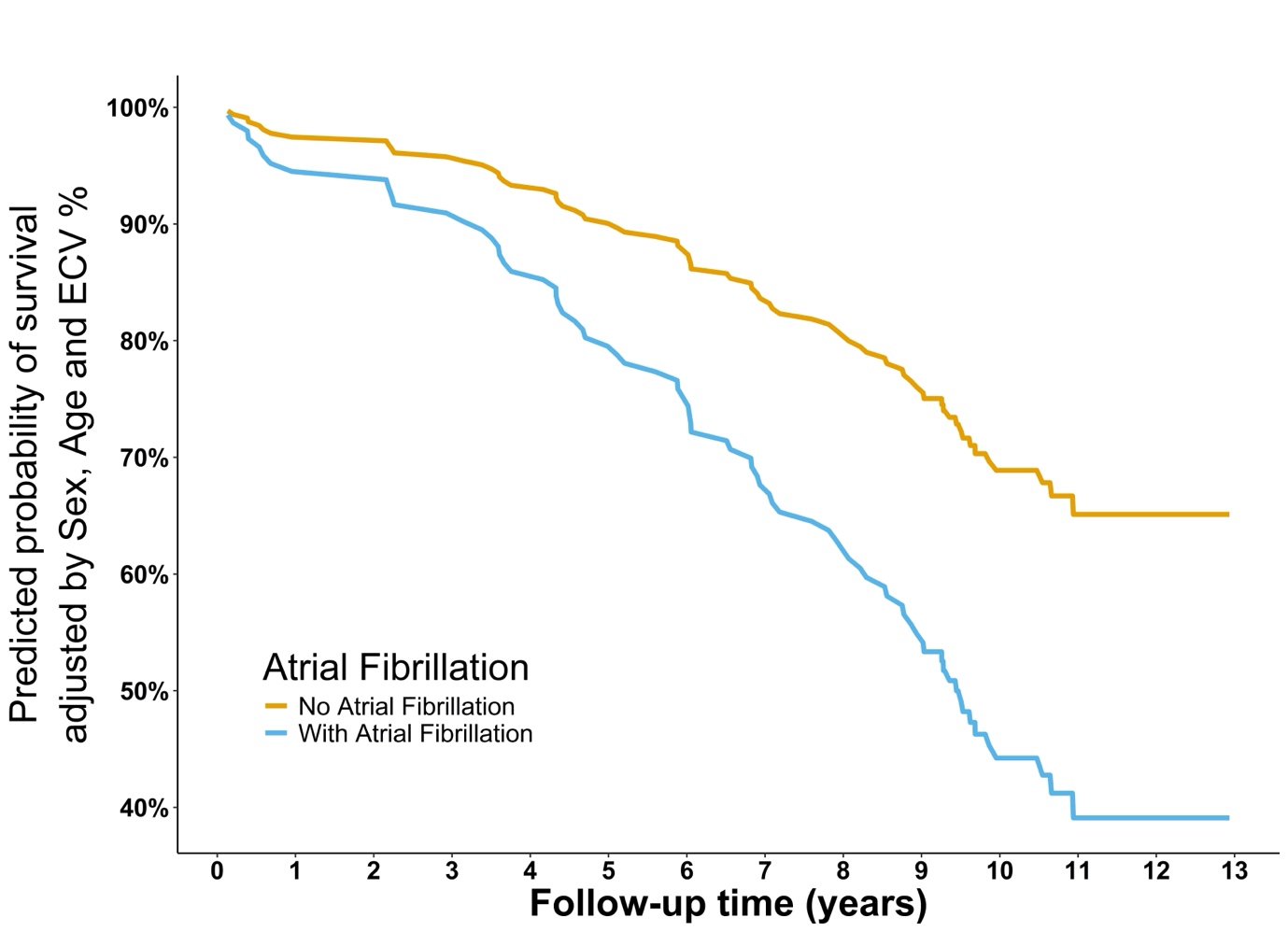

Supplement: Supplementary file 1 — Supplementary material [file mmc1.docx]
